# Enhanced angiogenesis, hypoxia and neutrophil recruitment during Myc-induced liver tumorigenesis in zebrafish

**DOI:** 10.1038/srep31952

**Published:** 2016-08-23

**Authors:** Ye Zhao, Xiaoqian Huang, Tony Weixi Ding, Zhiyuan Gong

**Affiliations:** 1 Department of Biological Sciences, National University of Singapore, Singapore 117543, Singapore

## Abstract

Angiogenesis, hypoxia and immune cells are important components in tumor microenvironment affecting tumor growth. Here we employed a zebrafish liver tumor model to investigate the effect of *Myc* expression on angiogenesis, hypoxia and tumor-infiltrated neutrophils during the tumor initiation stage. We found that induced *Myc* expression in the liver caused a dramatic increase of liver size with neoplastic features. The tumorigenic liver was accompanied by enhanced angiogenesis and inhibition of angiogenesis by an inhibitor (SU5416 or sunitinib) hindered the tumorigenic growth, suggesting an essential role of angiogenesis in tumorigenic growth of liver tumor in this zebrafish model. *Myc* induction also caused hypoxia, which could be further enhanced by hypoxia activator, ML228, to lead to a further enlargement of tumorigenic liver. Furthermore, *Myc* overexpression incurred an increase of liver-infiltrated neutrophils and the increase could be suppressed by angiogenesis inhibitors or by morpholino knockdown inhibition of neutrophil differentiation, leading to a suppression of growth of tumorigenic livers. Finally, the enhanced angiogenesis, hypoxia and tumor-infiltrated neutrophils by *Myc* overexpression were validated by RT-qPCR examination of expression of relevant biomarker genes. In sum, the current study demonstrated that the *Myc*-induced liver tumor model in zebrafish provides an excellent platform for study of tumor microenvironment.

*MYC* proto-oncogene encodes an important transcription factor that is involved in regulation of as many as 15% of cellular genes^1^. Overexpression of *MYC* has been found in various types of human cancers, including hepatocellular carcinoma (HCC), the most common type of liver cancers^2^. It has been found that aberrant *MYC* expression is often caused by genomic amplification and it is present in 70% of viral and alcohol-related HCC^3^. Other than promoting cell proliferation, the activation of tumorigenic *Myc* during hepatocarcinogenesis also causes changes in the tumor microenvironment by interacting with hypoxia-inducible factor-1 alpha (HIF-1α) and HIF-2α to increase angiogenesis^4^,^5^. Rapid proliferating tumor cells generally generate a mass which lacks oxygen (hypoxia) and this physical condition stabilizes HIFs to trigger a series of downstream gene expression, including genes for vascular endothelial growth factor (VEGF), platelet-derived growth factor (PDGF), fibroblast growth factor (FGF), angiopoietins and stromal derived factor-1α (SDF-1α)^6^, thus leading to angiogenesis. *MYC* has been found to post-transcriptionally induce HIF-1α protein and enhance HIF-1α accumulation under hypoxic conditions in cells^7^. Reciprocally, HIF-1α expression is functionally necessary for *MYC*-induced cell growth and proliferation^7^. *Myc* has been proven to be essential for vasculogenesis and angiogenesis, and loss of *Myc* impairs expression of *Vegf*^8^,^9^, suggesting a direct involvement of *Myc* in tumor angiogenesis. By analysis of human HCC specimens, it has also been found that HIF-1α expression correlates with inflammation, angiogenesis and *MYC* expression^10^.

Hypoxia stimulation could attract myeloid cells into the tumor microenvironment, which are then differentiated into tumor-associated macrophages or neutrophils and release cytokines, chemokines and proangiogenic growth factors to promote tumor progression^11^. Neutrophils are one of the most rapid responders of inflammatory cells to migrate towards the site of inflammation^12^. Recently, tumor associated neutrophils (TANs) were identified to be the key predisposing factor of tumor progression and angiogenesis^13^,^14^. By producing various cytokines and chemokines, TANs can influence the tumor cell proliferation, angiogenesis and metastasis^15^. The intracellular VEGF in neutrophils could be rapidly secreted upon stimulation and thus promotes angiogenesis by activating endothelial cells^16^,^17^. Neutrophil-derived matrix metalloproteinase-9 (MMP-9) has also been depicted to be responsible for VEGF release in the induction of angiogenesis in early stage of tumor growth in cancer models^18^,^19^. Moreover, upon recruitment to inflamed sites, neutrophils themselves can elicit hypoxia and modulate the host response to inflammation^20^. Thus, there is increasing evidence for the positive correlation among hypoxia, inflammation and angiogenesis^21^ and these three factors constitute important tumor microenvironment affecting tumor progression.

Our laboratory has previously generated an inducible liver tumor model in zebrafish, *TO*(*Myc*), by using a Tet-on inducible system to express mouse *Myc* oncogene with a liver-specific *fabp10a* promoter. With the induction of *Myc* expression by doxycycline (Dox), liver tumor was developed in adult zebrafish with essentially 100% penetrance^22^. The advantage of the inducible tumor model is the feasibility of investigation of tumor initiation as the timing of tumorigenesis can be controlled by addition of the chemical inducer; thus, this model should provide an important tool for investigation of changes of tumor microenvironment upon tumor initiation. In particular, the transparency of zebrafish embryos and availability of various fluorescence protein-targeted transgenic lines greatly facilitate the study of the interaction of different cell types in a tumor microenvironment. For example, a GFP reporter transgenic zebrafish line, *Tg*(*fli1:EGFP*), has EGFP specifically expressed in endothelia and it provides an excellent tool to visualize angiogenesis^23^. A hypoxia transgenic zebrafish line, *Tg*(*phd3: EGFP*), with EGFP expression driven by *prolyl hydroxylase 3* (*phd3*) promoter, has been established to modulate hypoxic response^24^. Another transgenic zebrafish line, *Tg*(*mpx: EGFP*), has neutrophils marked by GFP expression under the neutrophil-restricted *myeloperoxidase* (*mpx*) promoter^25^. As our inducible *TO*(*Myc*) transgenic line provides an excellent model to investigate the tumor initiation events, in this study, by crossing *TO*(*Myc*) with various reporter transgenic lines, the three tumor microenvironmental factors, angiogenesis, hypoxia and inflammation, were examined upon the induction of tumorigenesis by initiation of *Myc* overexpression. We observed an enhanced angiogenesis, hypoxia and neutrophil recruitment during liver tumor initiation.

## Results

### Increase of liver angiogenesis by overexpression of *Myc* oncogene in the liver

**Figure 1:**
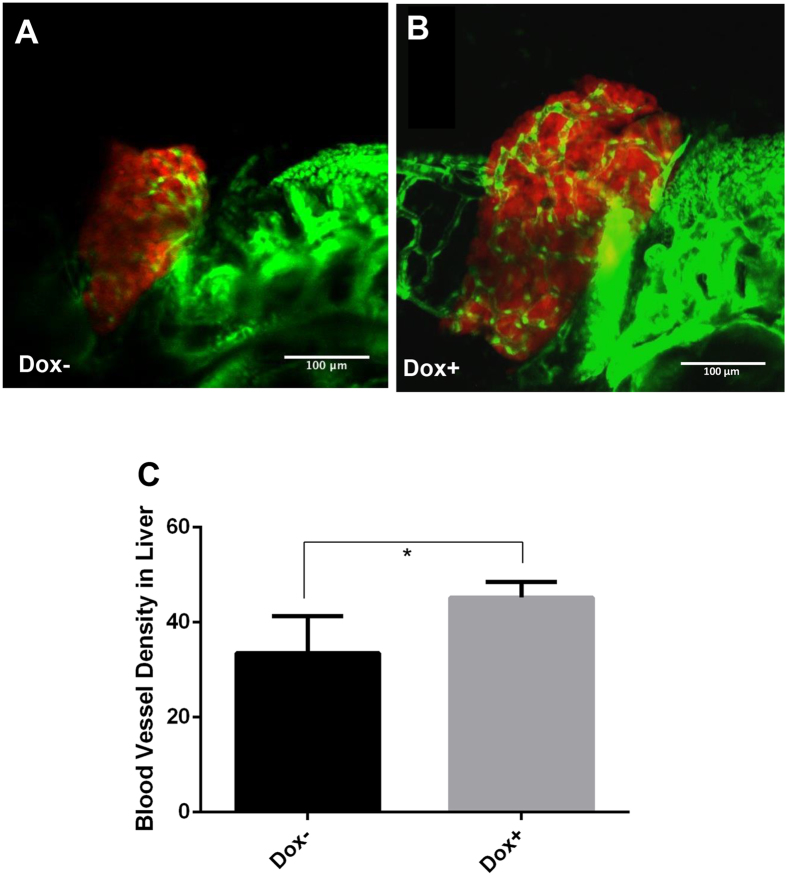
Enhanced angiogenesis in the liver by induction of transgenic *Myc* expression in *TO*(*Myc*) larvae. *Tg*(*Fli1:EGFP*)*, LiPan* and *TO*(*Myc*) zebrafish were crossed to generate triple transgenic zebrafish larvae. The liver was labeled by DsRed expression and blood vessel by EGFP expression. Triple transgenic larvae were treated with 30 μg/ml Dox from 3 dpf to 7 dpf and imaged by a confocal microscope. (**A**) A representative triple transgenic larva without Dox treatment. (**B**) A representative triple transgenic larva with Dox treatment. (**C**) Blood vessel density. Blood vessel density is defined by the ratio of blood vessel area (green) over the total liver area (as demarcated by DsRed expression) and measured by online ImageJ software. The quantitative data were based on 10 samples per group. The original magnification was 40x. Standard error bars are indicated and the two groups show significant difference with P-value < 0.05 by unpaired t-test statistical analysis. Scale bars = 100 μm.

To investigate angiogenesis in the *Myc*-induced zebrafish liver tumor model, *TO*(*Myc*) zebrafish were crossed with zebrafish of *Tg*(*fli1:EGFP*) and *Tg*(*fabp10:RFP,ela3l:EGFP*) (known as LiPan with Ds-Red expression in the liver and EGFP expression in the exogenous pancreas)^26^ to produce triple transgenic larvae in order to visualize both liver (Ds-Red expression) and blood vessels (EGFP expression). Confocal microscopy was used to produce Z-stack images of the vascularization in the triple transgenic larvae. As shown in Fig. 1, there was a significant increase of angiogenesis, as indicated by EGFP-labeled blood vessels in Dox-induced larvae (Fig. 1B) compared to non-induced larvae (Fig. 1A). Quantitation was made by measuring the ratio of the green area (blood vessels) over the entire liver area using Image J software. In addition to the enlargement of liver as described in our previous studies^22^, the blood vessel density was also significantly higher upon Dox induction than that without Dox induction (Fig. 1C); thus, *Myc*-induced tumorigenesis was associated with increased angiogenesis.

**Figure 2:**
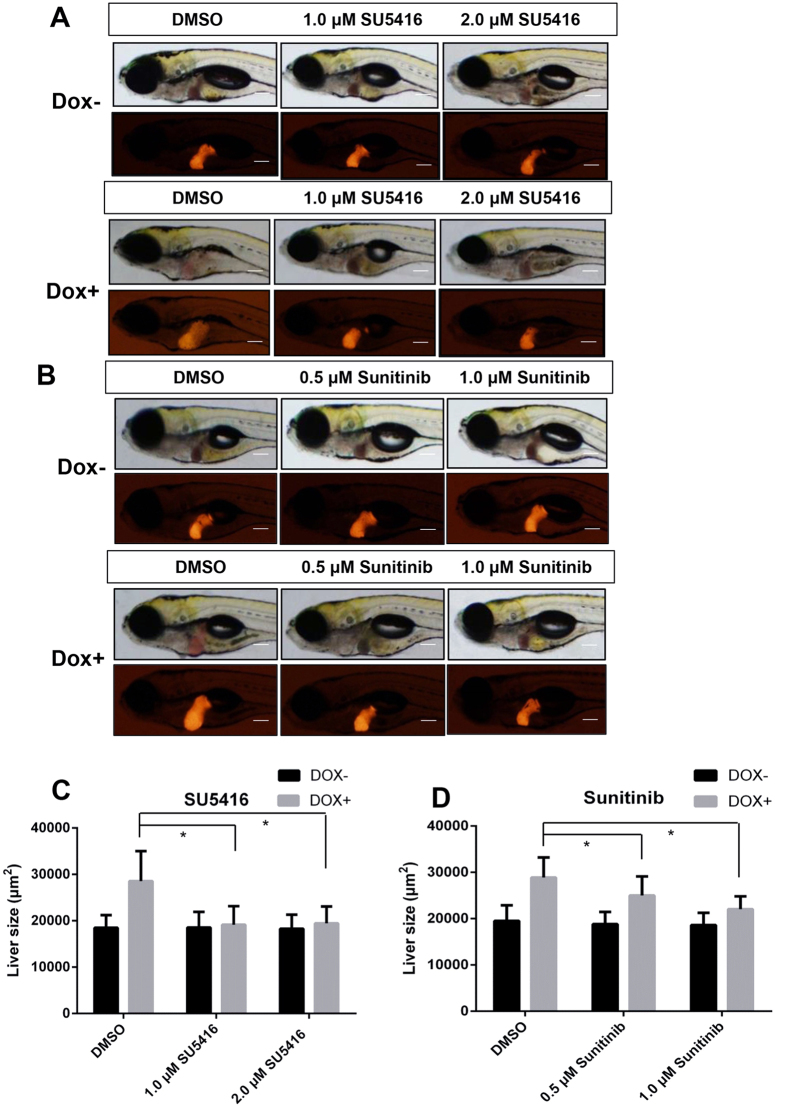
Effects of angiogenesis inhibitors on tumorigenic liver growth. *To*(*Myc*)*/LiPan* double transgenic larvae were treated with anti-angiogenesis compounds SU5416 (1 μM or 2 μM) or sunitinib 0.5 μM or 1 μM) with or without 30 μg/ml Dox from 3 dpf to 7 dpf. 0.1% DMSO was used as vehicle control for both compounds. Liver areas were imaged and 2D liver areas were quantified. (**A,B**) Images of representative 7-dpf double transgenic larvae treated with different concentrations of SU5416 (**A**) and sunitinib (**B**). Both bright field (top) and fluorescent images (bottom) are shown. (**C,D**) Quantification of changes of 2D liver size after treatment with SU5416 (**C**) and sunitinib (**D**). The quantitative data were based on 10 samples per concentration group. The original magnification was 10x. Data are represented as mean ± SD. Astrisks indicate significant difference with P-value < 0.05 by two way ANOVA statistical analysis. Scale bars = 100 μm.

To demonstrate the role of angiogenesis in *Myc*-induced tumorigenesis, two angiogenesis inhibitors, SU5416^27^ and sunitinib^28^, were used to treat the larvae together with Dox. The treatments were conducted for *LiPan/Myc* double transgenic larvae from 3 dpf to 7 dpf in two concentration groups for each drug: 1.0 μM and 2.0 μM for SU5416; 0.5 μM and 1.0 μM for sunitinib. All treated larvae survived under these concentrations throughout the experiment duration. Fluorescent images were taken and representative images are shown in Fig. 2A,B. Liver sizes were measured based on 2D outline of Ds-Red labeled livers as previously described^22^,^29^. As summarized in Fig. 2C,D, there was a significant increase of liver size upon Dox induction in the absence of angiogenesis inhibitor (0 μM groups). However, in the presence of either inhibitor, liver enlargement was significantly suppressed in both tested concentrations (1 and 2 μM for SU5416; 0.5 and 1 μM for sunitinib). Thus, angiogenesis is apparently required for tumorigenic liver growth upon *Myc* induction.

### Induction of liver hypoxia during *Myc*-induced liver tumorigenesis

**Figure 3:**
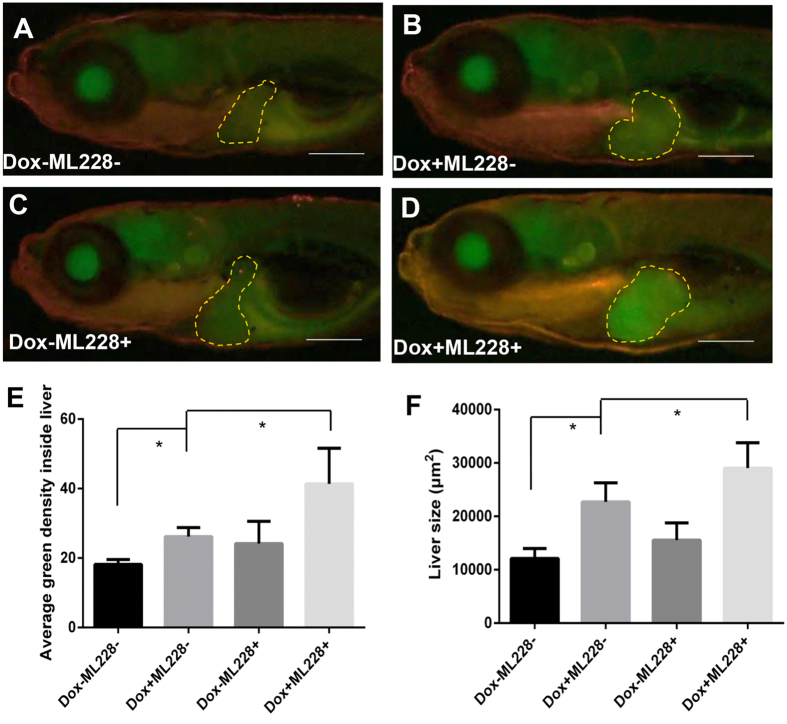
Stimulation of tumorigenic liver growth by hypoxia. *TO*(*Myc*) and *Tg*(*phd3::EGFP*) double transgenic larvae were generated and induced by Dox for 4 days from 4 dpf to 7 dpf. (**A–D**) Images of liver hypoxia as indicated by EGFP expression in dashline-circled liver areas. A non-Dox treated control is shown in (**A**) and a Dox treated larva is in (**B**). 0.5 μM ML228 was used to enhance hypoxia and representative images are shown in (**C,D**) in the absence or presence of Dox, respectively. The original magnification was 20x. (**E**) Quantification of level of hypoxia as indicated by average GFP green density inside the liver. (**F**) Quantification of 2D liver size. The quantitative data were based on 5 samples per concentration group. Data are represented as mean ± SD. Astrisks indicate significant difference with P-value < 0.05 by unpaired t-test statistical analysis. Scale bars = 100 μm.

It has long been known that hypoxia is a key regulator of angiogenesis during tumor progression, in which the activation of hypoxia-related genes (e.g. *Hif1α*) would result in the activation of angiogenesis-related pathways and thus lead to increase of blood vasculature^30^. Recently it has also been reported that *Myc* plays a role in enhancing the stability of the hypoxia-inducible factor Hif1α in tumorigenic cells^31^. To verify the status of hypoxia in our *Myc* tumor model, a hypoxia reporter transgenic line *Tg*(*phd3:EGFP*) was crossed with *TO*(*Myc*) and the double transgenic larvae were induced with Dox for 4 days from 3 dpf and imaged under a fluorescent microscope, where the hypoxia status was indicated by the intensity of green fluorescence. As shown in Fig. 3, the level of EGFP expression was increased significantly in Dox-treated larvae compared to non-treated controls (Fig. 3A,B,E), indicating the presence of hypoxia in *Myc*-induced tumorigenic livers. To further validate the hypoxia status in *Tg*(*phd3: EGFP*) larvae, a hypoxia activator ML228 (0.5 μM) was used to further activate the Hif pathway^32^ and indeed a further increase of the EGFP expression in the liver was observed (Fig. 3C–E). Importantly, the addition of ML228 also conferred a further increase of the tumor size (Fig. 3F), suggesting that hypoxia activation could further accelerate tumor growth in our transgenic model.

### Enhanced liver recruitment of neutrophils during *Myc*-induced liver tumorigenesis

**Figure 4:**
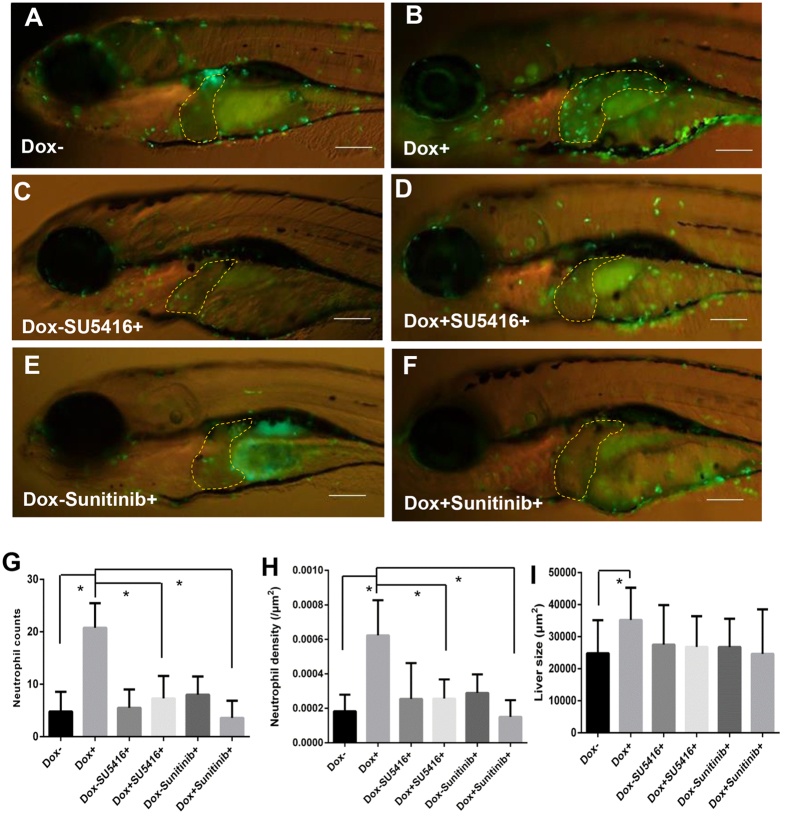
Enhanced tumor infiltration of neutrophils by induced *Myc* expression and suppression of neutrophil infiltration by angiogenesis inhibitors. *TO*(*Myc*) and *Tg*(*mpx:EGFP*) double transgenic larvae were generated with EGFP labeled neutrophils. (**A–F**) Images of the double transgenic larvae in the presence of the following chemicals: nil control (**A**), Dox (**B**), SU5416 (**C**), Dox+SU5416 (**D**), sunitinib (**E**) and Dox+sunitinib (**F**). The larvae were treated from 4 dpf to 7 dpf. In all images, the liver areas are circled by dashlines. The original magnification was 20x. (**G**) Neutrophil counts in the liver. (**H**) Neutrophil density in the liver. Neutrophil density was calculated as number of neutrophils per μm^2^. (**I**) Quantification of 2D liver size. The quantitative data were based on 10 samples per concentration group. Data are represented as mean ± SD. Astrisks indicate significant difference with P-value < 0.05 by unpaired t-test statistical analysis.

Neutrophils are the most abundant immune cells in the innate immune system and also the first responders when acute inflammation occurs. In some cancers, neutrophils have been found to promote tumor development^34^,^35^, including the *kras*-induced zebrafish liver cancers we recently reported^36^. In this study, in order to investigate the behavior of neutrophils in *Myc*-induced liver tumorigenesis, we crossed *TO*(*Myc*) with *Tg*(*mpx:EGFP*) transgenic zebrafish. After induction by Dox from 3 dpf to 7 dpf, there was a rapid increase of neutrophil infiltration in the tumorigenic livers (Fig. 4A,B). Interestingly, the increase of neutrophil infiltration could be suppressed by either inhibitor of angiogenesis, SU5416 or sunitinib (Fig. 4C–F). Quantification of the neutrophil counts (Fig. 4G) and the density of neutrophils in the livers (Fig. 4H) also confirmed the increase of neutrophil recruitment by Dox induction and suppression by angiogenesis inhibitors. Quantification of the liver size (Fig. 4I) was also consistent with the angiogenesis inhibitor experiment as shown in Fig. 2.

**Figure 5:**
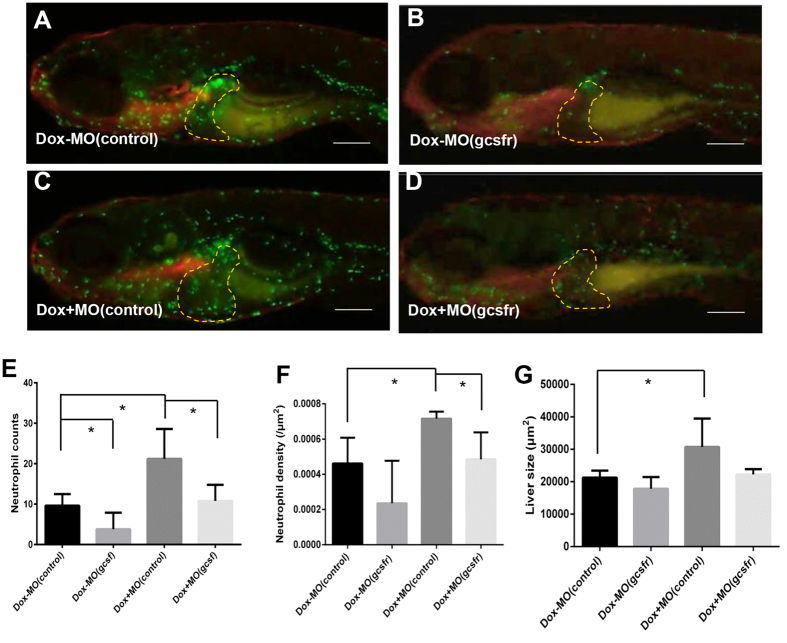
Stimulation of tumorigenic liver growth by tumor-infiltrated neutrophils. *TO*(*Myc*) and *Tg*(*mpx:EGFP*) double transgenic larvae were used for norpholino knockdown of Gcsfr to inhibit neutrophil differentiation. Morpholino oligonucleotides were injected into the embryos at 1–2-cell stage. (**A–D**) Images of the double transgenic larvae after injection of either MO(control) (**A,C**) or MO(gcsfr) (**B,D**). These injected embryos were either treated with Dox (**C,D**) or without Dox from 4 dpf to 7 dpf (**A,B**). The liver areas are circled. The original magnification was 20x. (**E**) Neutrophil counts in the liver. (**F**) Neutrophil density in the liver under different conditions. (**G**) Quantification of 2D liver size. The quantitative data were based on 5 samples per concentration group. Data are represented as mean ± SD. Asterisks indicate significant difference with P-value < 0.05 by unpaired t-test statistical analysis. Scale bars = 100 μm.

To further analyse the role of neutrophils in tumorigenic liver tumor growth, morpholino knockdown of Gcsfr was performed as previously described and validated in the zebrafish larvae within 7 days old^36^. As shown in Fig. 5, the number of neutrophils infiltrated to the liver was significantly decreased compared to controls injected with MO(control) (Fig. 5A,B). After Dox induction, neutrophils were increased as expected in the MO(control)-injected group (Fig. 5C); however, in the MO(gcsfr)-injected group, the number of neutrophils in the liver region was significantly dropped compared to the Dox/MO(control) group (Fig. 5D). The increase and decrease of neutrophils were confirmed by quantification of neutrophil counts and density in the four experimental groups (Fig. 5E,F). Interestingly, the liver size correlated to the density of neutrophils in the four groups, suggesting that the recruitment of neutrophils may play a stimulating role during *Myc*-induced liver tumor initiation (Fig. 5G).

### Upregulation of genes for angiogenesis, hypoxia and inflammation during *Myc*-driven liver tumorigenesis

**Figure 6:**
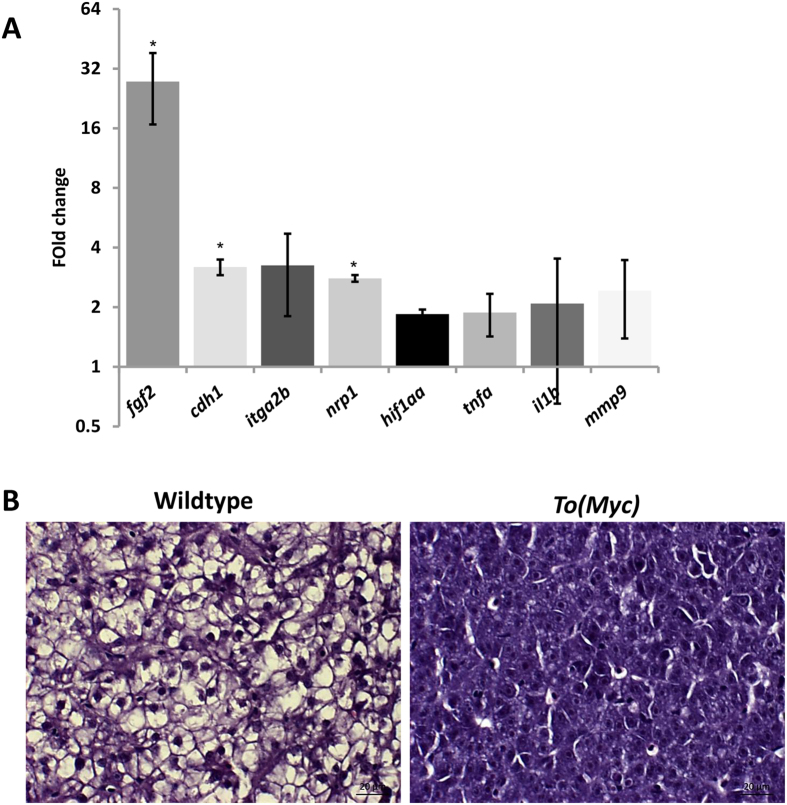
Molecular and histological characterization of Myc overexpressed livers. One-month-old wildtype or *TO*(*Myc*) zebrafish were treated with 30 μg/ml Dox for 7 days and euthanized for RNA extraction and histological analyses. (**A**) Validation of increased angiogenesis, hypoxia and inflammatory response by biomarker gene expression. RNA expression of selected biomarker genes were measure by RT-qPCR. Fold changes shown are ratio of the values from *To*(*Myc*) fish over wildtype fish after calibration with beta-actin mRNA as an internal control. Asterisks indicate significant difference with P-value < 0.05 by t-test among the three biological replicates. (**B**) Histological comparison of livers from wildtype (left) and *TO*(*Myc*) (right) fish. Fish were treated with or without Dox (30 μg/ml) from 4 dpf to 7 dpf. Representative pictures are shown for each group (n = 10 per group). The original magnification was 100x. Scale bars = 20 μm.

As demonstrated in Figs 1, 2, 3, 4 and 5, induction of *Myc* expression had stimulating roles in angiogenesis, hypoxia and neutrophil recruitment. To verify these events at molecular level, selected genes were examined by RT-qPCR for their expression after induction of *Myc* expression in the livers of one-month-old juvenile *TO*(*Myc*) zebrafish treated with Dox for 7 days. As shown in Fig. 6A, angiogenesis related factors *fgf2* (fibroblast growth factor 2)^37^, *cdh1* (cadherin 1)^38^ and *itga2b* (*integrin α2b*)^39^ were all up-regulated compared with the group without Dox treatment, confirming that angiogenesis pathway was activated in *Myc*-induced liver tumors. Similarly, hypoxia biomarker gene, *hif1aa*^40^, and inflammation-related genes such as *nrp1* (*neurophilin 1*)^41^, *tnfα* (tumor necrosis factor *α*)^42^, *il1b* (interleukin 1b)^43^ and *mmp9* (matrix metallopeptidase 9)^44^ were also up-regulated, further confirming the positive roles of hypoxia and inflammatory responses during *Myc*-induced liver tumorigenesis.

Previously we have reported that hyperplasia can be induced in *TO*(*Myc*) fish within 3 weeks of Dox induction while hepatocellular carcinoma induced within 16 weeks of Dox induction despite that strong molecular signature for HCC has been discovered^22^. In another transgenic zebrafish modal using zebrafish *myc* oncogene, HCC can be observed within six months of *myc* induction^45^. To confirm that tumorigenesis after *Myc* induction in this study, one-month-old juvenile *TO*(*Myc*) fish after 7 days of Dox induction were histologically analysed. We found that neoplastic changes could be observed within 7 days of Dox induction. As shown in Fig. 6B, *Myc* induced liver showed early neoplastic features, including disrupted arrangement of hepatic plates, an increase number of hepatocytes (hyperplasia), enlargement of nuclei and nucleoli and reduced glycogen deposit. The detailed characterization of newly induced liver tumors in *TO*(*Myc*) fish is in preparation for publication separately.

## Discussion

*Myc* proto-oncogene is a prominent driver in development of liver cancers and several *Myc* transgenic animal models for tumorigenesis have been generated in mice^45^,^46^. Recently we have generated several inducible liver tumor models by transgenic expression of either mouse *Myc*^22^ or zebrafish *myc* genes^44^. Other than the well-recognized attributes of the zebrafish model such as *in vitro* development and availability of a large number of embryos/larvae, our zebrafish liver tumor models offer several advantages over other animal models. First, there are a large number of fluorescent protein transgenic zebrafish targeting different cell types and these transgenic zebrafish could be easily bred with the liver tumor transgenic lines for detailed investigation of interaction of different cells in a tumor microenvironment. In particular the transparency of zebrafish embryos/larvae provides excellent tools for applying advance bioimaging technologies to investigate interaction of different types of cell in real-time and in a more precise manner^47^. Second, pharmacological intervention could be easily conducted by immersion exposure of zebrafish including embryos/larvae to small molecules in a small volume and thus to study the biological functions disrupted. Finally, our inducible liver tumor models allow us to time tumorigenesis and thus it is feasible to characterize the tumor initiation events. In the present study, we have taken all of these advantages to demonstrate the accessibility of the three major components of tumor microenvironments (angiogenesis, hypoxia and tumor-infiltrated neutrophils) and demonstrated that all the three factors have promoting effects on tumorigenic growth of the liver as inhibition of any one of these factors caused hindrance of the growth of tumorigenic livers.

Angiogenesis is deemed necessary in *Myc*-induced tumorigenesis since angiogenesis inhibitors could reduce tumorigenic liver growth (Fig. 2). This observation is consistent with observations from a zebrafish xenograft model^48^, in which inhibition of tumor angiogenesis could also significantly decrease VEGF-induced tumor cell dissemination and metastasis. Because of the transparency of zebrafish embryos/larvae and the feasibility of observing angiogenesis in live imaging by using *Tg*(*fli1:EGFP*) transgenic zebrafish, to date, many cancer xenotransplantation zebrafish models have been reported for tumor-associated angiogenesis studies and for high-throughput drug screenings^49^,^50^,^51^,^52^,^53^,^54^,^55^. It is suggested that anti-tumor-associated angiogenesis has proven to be a new therapeutic strategy. To our knowledge, the *Myc* transgenic zebrafish liver angiogenesis model in our research is the first transgenic zebrafish liver tumor angiogenesis model for anti angiogenic drug research. Indeed, overexpression of *Myc* is essential to regulate tumor-mediated angiogenesis and tumor growth^8^. *Myc* promotes vascular and hematopoietic development by functioning as the main regulator of angiogenetic factors^56^. In particular, *Myc* could interact with hypoxia to enhance angiogenesis by a VEGF-dependent mechanism^57^. In this study, we have taken advantage of transparency of zebrafish embryos to demonstrate the increased angiogenesis in *Myc*-induced zebrafish liver tumors. The induced liver tumors could be reduced by pharmacological treatments, thus indicating the feasibility of development of a high throughput screening platform using our zebrafish liver tumor models for discovery novel anti-cancer drugs that targeting inhibition of angiogenesis.

Hypoxia has been proven to play a prominent role in inducing angiogenesis in abnormal tumor vascularization and metastasis^58^. The prolyl 4-hydroxylase domain proteins (PHDs) act as dual enzymes. With sufficient oxygen supply, PHDs regulate HIF-α in von Hippel-Lindau protein (pVHL)-mediated proteasomal destruction. Oxygen availability plays a major role in PHD-catalyzed activities^59^,^60^. Under prolonged hypoxia situation, including tumor induced hypoxia, the adaptive oxygen homeostasis induces overactivation of PHDs to mediate HIFα desensitization, which ensure cell survival and proliferation^61^. Among three PHDs isoforms (PHD1, PHD2 and PHD3), PHD3 shows the strongest up-regulation under hypoxia^60^. Using the *phd3* promoter, Kirankumar *et al*. have generated a transgenic line, *Tg*(*phd3:EGFP*), in which EGFP expression is triggered indicating Hif activation and thus the transgenic line could be used as a live reporter for tracking hypoxia *in vivo* in zebrafish^24^. By using the *Tg*(*phd3:EGFP*) line, in our study, we also verified that the hypoxia was induced in *Myc*-transgenic zebrafish during tumorigenesis and the activation of hypoxia could accelerate tumorigenesis by promoting liver overgrowth (Fig. 3). The hypoxia enhancer ML228 is a newly discovered activator of the HIF pathway, which takes part in the angiogenesis processes initiated by lower oxygen availability in the bloodstream^32^. By using ML228 in our research, we have further illustrated induced hypoxic situation could accelerate tumor growth in our transgenic model. It has been reported that in tumor microenvironment, angiogenesis is driven by hypoxia via VEGF and other pro-angiogenic factors^62^ and that hypoxia or overexpression of HIF-1a could regulate the expressions of Twist to promote epithelial–mesenchymal transition and thus increase metastasis^63^. Hypoxia could also activate Stat3^31^ and Notch signaling pathway^64^, both of which have important roles in promoting tumor development. In addition to stimulate angiogenesis, fibrosis and liver carcinogenesis, hypoxia could also play an aggravating role in cell damage and inflammation^65^. Collectively, tumor induced hypoxia favors tumor cell proliferation and thus could be a useful target for cancer therapy.

Previously we have reported that tumor-infiltrated neutrophils play a stimulating role in *kras*-induced liver tumorigenesis in another zebrafish model^35^. Now we have found a similar role of tumor-infiltrated neutrophils in *Myc*-induced liver tumorigenesis; thus, it is likely that tumor-infiltrated neutrophils have a generally stimulating role in liver tumors and probably also in other tumors. In the present study, morpholino approach was used to suppress neutrophil differentiation, but it is difficult to achieve a complete elimination of neutrophils by this approach. In future, inducible ablation of neutrophils could be achieved by developing novel transgenic lines as previously reported^66^,^67^. Neutrophils play a key role in mediating tumor angiogenesis as well as hypoxia in tumor microenvironment^21^. Our findings directly link tumor-associated neutrophils to tumor angiogenesis. Inhibition of angiogenesis could reduce the recruitment of neutrophils into the tumorigenic liver and inhibit liver overgrowth (Figs 4 and 5). Previously it has also been reported that inhibition of angiogenesis in tumor cell transplanted zebrafish larvae did not reduce the number of neutrophils but increase the speed of random migration, which may in turn promoter tumor cell invasion^50^. This experiment could be further carried out in the current inducible zebrafish HCC model in future. The interplay between inflammation (neutrophils) and angiogenesis can be executed via matrix metalloproteinases (MMPs), which are stimulated to release angiogenetic factors into the extracellular matrix^11^,^15^. Neutrophil-derived MMPs, in particular MMP9, have been shown to be the most important activator in tumor vascularization^43^. Consistent with this, *mmp9* was also found to be up-regulated in Myc-induced liver tumorigenesis in this study (Fig. 6A). Hypoxia and inflammation have also been reported to have an interdependent relationship, where hypoxia could lead to secondary inflammatory changes^68^, and inflammation could in turn stabilize HIF^69^. Our current data are consistent with these observations, thus demonstrating the usefulness and effectiveness of our *Myc* transgenic zebrafish model in studying tumor microenvironment.

There is growing evidence that anti-angiogenic treatments can transiently normalizes tumor vessels, The therapeutic effect is transitory and is ultimately followed by active tumor angiogenesis as tumors rapidly adapt to the effects of anti-VEGF agents and induces refractoriness^70^,^71^. Hypoxia and HIF-dependent responses play an essential role in several of these adaptive signals^72^,^73^. Recruitment of myeloid cells including neutrophils is also associated with these responses^74^. In addition, the migration of neutrophils can be enhanced by anti-VEGF agents, which contributes to tumor invasion and micrometastasis^50^. In the present study, our efforts were limited to the tumor initiation phase and we tested only the transient effects of the anti-angiogenic compounds. It will be interesting in future to test these during long term tumor progression in the current Myc-induced HCC model in zebrafish and investigate the combination therapy of anti-VEGF treatment with the inhibition of myeloid cells.

## Methods

### Zebrafish maintenance

All zebrafish experiments were carried out in accordance with the recommendations in the Guide for the Care and Use of Laboratory Animals of the National Institutes of Health and the protocol was approved by the Institutional Animal Care and Use Committee (IACUC) of the National University of Singapore (Protocol Number: 096/12). *TO*(*Myc*) (gz26Tg) and *LiPan* (gz15Tg) were generated from our own lab: *To*(*Myc*) contains three co-integrated transgenic constructs, *Tg*(*fabp10a:RTTA;TETRE:Mmu.Myc;krt4:RFP*), to have Dox-induced mouse *Myc* expression plus constitutive skin EGFP expression for transgenic identification^22^; *LiPan* contains two co-integrated constructs, *Tg*(*fabp10:RFP,ela3l:EGFP*), and has RFP (Ds-Red) expression in the liver and EGFP expression in the exogenous pancreas^26^. *Tg*(*fli1:EGFP*) (y1Tg)^23^, *Tg*(*phd3:EGFP*)) (sh144Tg)^24^ and *Tg*(*mpx:EGFP*) (rj30Tg)^25^ were obtained from Drs. B. M. Weinstein, E. van Rooijen and S.A. Renshaw, respectively.

### Chemical treatments

Doxycycline (Dox), SU5416 (1, 3-Dihydro-3-[(3,5-dimethyl-1H-pyrrol-2-yl)methylene]-2H-indol-2-one), Sunitinib (Sunitinib malate) and ML228 (N-([1, 1′Biphenyl]-4-ylmethyl)-6-phenyl-3-(2-pyridinyl)-1, 2, 4-triazin-5-amine) were purchased from Sigma-Aldrich. Chemical treatments were conducted in 6-well plates for zebrafish larvae from 3 dpf to 7 dpf and in 1-L tanks for juvenile fish.

### Histological analyses

Fish samples were fixed with formalin solution (Sigma-Aldrich). Five-micrometer sections were processed using a microtome and stained with hematoxylin and eosin.

### Microscopy

To facilitate visualization of livers in live zebrafish larvae, skin pigmentation was inhibited using 0.2 mM 1-phenyl-2-thiourea (Sigma, USA)^75^. Microscopic observations and photography of live larvae were performed using a dissecting fluorescent microscope (Olympus SZX12, Japan), a compound microscope (Zeiss Axioscope 2, Germany) and a confocal microscope (Zeiss LSM510). The liver areas were quantified by online Image J software as previously described^22^,^29^.

### RNA isolation and RT-qPCR

Liver samples were collected from 1 month juvenile fish treated with Dox for 4.5 days. Total RNA was isolated using TriZOL reagent (Invitrogen) and reverse-transcribed using the SuperScript II cDNA Synthesis Kit (Invitrogen). RT-qPCR was performed using LightCycler^®^ 480 SYBR Green I Master system (Roche). Each sample was analysed in triplicate. Βeta-actin mRNA was used as the internal control for calibration of gene expression levels between samples. Log2 fold changes between tumor (Myc+ with Dox treatment) and control (wildtype fish with Dox treatment) samples were calculated by the −∆∆CT method^76^. Primers used for PCR are shown in Table 1.

### Mopholino knockdown

Inhibition of neutrophil differentiation was performed by knockdown of Gcsfr (granulocyte colony-stimulating factor receptor) using a previously validated morpholino oligonucleotide targeting a splice site, MO(gcsfr) (5′-GAAGCACAAGCGAGACGGATGCCAT-3′)^35^,^77^. A standard control morpholino oligoneucleotide targeting a human beta-actin intron, MO(control) (5′-CCTCTTACCTCAGTTACAATTTATA-3′), was also used. Both morpholino oligonucleotides were synthesized by Gene Tools (USA). A total of 1 nL of 1 mM morpholino oligonucleotide was injected *Tg*(*mpx: EGFP*)*/TO*(*Myc*) double transgenic embryos at 1–2 cell stage.

### Statistical analyses

Statistical analyses were performed by two-tailed unpaired t tests and two way ANOVA tests using inStat version 5.0 for Windows (GraphPad, San Diego, CA). Data in bars represent mean ± s.d in histograms. Zebrafish larvae for measurement were randomly collected in all experiments and differences were considered statistically significant at P < 0.05.

## Additional Information

**How to cite this article**: Zhao, Y. *et al*. Enhanced angiogenesis, hypoxia and neutrophil recruitment during Myc-induced liver tumorigenesis in zebrafish. *Sci. Rep.* **6**, 31952; doi: 10.1038/srep31952 (2016).

## Figures and Tables

**Table 1:** PCR primer sequences.

Genes	Forward Primers	Reverse Primers
*β-actin*	CCACCTTAAATGGCCTAGCA	CATTGTGAGGAGGGCAAAGT
*fgf2*	GGAGGAAAACCACTACAACAC	ACCTGTCGTGGAAGAAAGAAAATGG
*cdh1*	AGTGCCTCTTGACTATGAA	CCCTCGAACACGCTAAAC
*integrin a-2b*	GTAACTGCTGTCGCTTCC	CGGTTCCTTAGCTCATAT
*neurophilin 1*	TTCAGTCCAGTGGCACCC	CAGAAAGACAGGCAGAGG
*hif1aa*	GATGCTCGTCCACAGAAC	CACGTCGCAGACTTGATA
*tnfa*	TCAAAGTCGGGTGTATGG	CCTGGTCCTGGTCATCTC
*il1b*	CCCCAATCCACAGAGTTT	AGCTGGTCGTATCCGTTT
*mmp9*	TCGGGAAACTAGATCACGG	GGTCCAGAGCCAAGGGTC
